# Teaching psychiatry to large groups in society

**DOI:** 10.1186/s12909-019-1596-9

**Published:** 2019-05-16

**Authors:** Susanne Bejerot, Ann Lindgren, Jörgen Rosén, Eva Bejerot, Marie Elwin

**Affiliations:** 1 School of Medical Sciences, Örebro University, Örebro, Sweden; 2 University Health Care Research Center, Faculty of Medicine and Health, Örebro University, Örebro, Sweden; 3 Central Health Services in pre-schools, schools and upper secundary schools, Municipality of Norrtälje, Stockholm, Sweden; 4 Department of Psychology, Uppsala University, Uppsala, Sweden; 5 The Örebro University School of Business, Örebro, Sweden

**Keywords:** Patient-centered care, Staff development, Mental disorder, Intellectual disability, Health education, Social support, Mental health services, Nidotherapy

## Abstract

**Background:**

There is a need to educate a range of professionals in caring for individuals with long-term mental disability who reside within our communities. Empathy alone is insufficient. The Kognus 4-Step Education Program was developed to achieve this goal.

**Method:**

The program consisted of independent courses, including an 18-session basic course on psychiatric disability (on-site or online), advanced courses, and highly specialized training programs (Nidotherapy/Peer Consultation). Experts lectured together with clients with psychiatric disabilities. We first report Swedish reforms in which institutionalized patients were relocated to semi-independent individual households. We then describe the design and implementation of the education program. Approximately 50% of participants who were younger than 36 years old lacked any healthcare education. The participants’ backgrounds, perceptions, participation in the education program, and costs are presented.

**Results:**

Between 2009 and 2014, 8959 participants attended the Kognus psychiatry courses online or on-site in Stockholm (basic on-site course, *n* = 2111; online course, *n* = 4480; advanced courses, *n* = 2322; highly specialized programs, *n* = 46). A total of 73% of the participants satisfactorily attended the basic sessions on-site compared with 11% of the online participants. The developers conducted the education program for the first 3 years. Thereafter, another course provider continued the program with other types of participants. The program was perceived to be equally interesting and meaningful to participants with low and high levels of education, demonstrating the generalizability of the program. The quality of the basic and advanced courses was rated as 4.4 and 4.3, respectively, on a 5-point Likert scale.

**Conclusions:**

Personnel without appropriate education who work with people with psychiatric/intellectual disabilities can be educated in large numbers. The Kognus program represents a novel and successful way of training people who have no formal education about some essentials of good mental healthcare. Moreover, the model can be easily implemented elsewhere.

**Electronic supplementary material:**

The online version of this article (10.1186/s12909-019-1596-9) contains supplementary material, which is available to authorized users.

## Background

Assisting people with psychiatric and intellectual disabilities is difficult without adequate knowledge—empathy alone is insufficient. In this report, we present an education program that consists of independent courses that are based on a patient-centered approach. In person-centered care, the patient’s informed voice is imperative, and individual preferences, needs, and values are respected. The care is provided in an empathic and respectful way [1]. Client participation and the use of client narratives in healthcare education have been shown to promote a person-centered approach [2]. Such an approach was used throughout the planning and implementation of the Kognus project. The courses are aimed at professionals with various occupational backgrounds who encounter and care for these individuals in their everyday work.

This report includes an introduction on the ways in which the care of psychiatric patients in Sweden has developed through a number of reforms over the years. The specific aim of these reforms is to provide psychiatric patients with the possibility of living a normal life within society. A similar shift in the mental health system from hospital-based care to community-based care has occurred in other economically developed countries [3]. We report the reasons why the education of psychiatric personnel has declined in this process. We also report why the Kognus education program was introduced and the ways in which it was designed, implemented, and eventually transferred to other educational providers. Lastly, we present the participants’ satisfaction with the program.

Four major changes that are related to implementing the Community Mental Health Care Reform in 1995 [4] occurred during the last 20 years in Sweden. These changes impacted the educational needs of mental healthcare workers. First, the permanent care of patients in mental hospitals was replaced with long-term community-based residential services and daycare services, including occupational and vocational rehabilitation. The goal was to provide opportunities to live independently and integrate into the local community. Since 1991, 70% of all hospital beds in Swedish psychiatric wards have disappeared [5]. Although this is a positive trend, there is a correlation between the closing of mental hospitals and higher mortality rates among psychiatric patients [5–8].

Community-based mental health services have employed homecare assistants, a new profession in Sweden that requires no formal education or training, to help patients in their home lives. The division of responsibility between community-based care and hospital-based psychiatric services has often resulted in disagreements that have adversely affected such collaborations and may harm the client.

The second change that was caused by the reform of 1995 involved the field of intellectual developmental disorders [9]. Primary care doctors would henceforth manage these patients and treat them no differently from any other patient. This was based on a desire to normalize the lives of intellectually disabled individuals. However, co-occurring medical conditions are common in people with intellectual disabilities [10–12], and their treatment outcomes are more difficult to evaluate than those who are intellectually able [13–15]. Misjudgments and inappropriate treatments for intellectually disabled patients have emerged over the years [16].

The third important change was the termination of free state-administered education programs for mental healthcare assistants that had been running since 1967 [17]. Accordingly, people with inadequate or poor educational backgrounds often carry the responsibility of caring for chronically ill psychiatric patients.

The fourth change is related to psychiatric morbidity in the general population, which is the most common reason for sick leave in Sweden [18]. Between 2010 and 2015, sick leave in Sweden increased by 80, and 59% of this increase was attributable to psychiatric morbidity [19]. This increase in both sickness-related absence and incapacity benefits has been a major concern in most high-income countries [20]. State initiatives to curb insurance spending through more stringent assessments of sick leave records have resulted in a greater number of rejections of claims. Many patients who are unable to work are left without disability benefits [21]. The Swedish expert authority for social security gave directives to job agencies that they should actively coach long-term unemployed individuals, including people with mental disability, to find work [22]. New professional groups within social insurance and employment services need to be educated about the problems and disabilities that they may encounter with these clients.

In conclusion, increasingly more adults with severe psychiatric disorders or intellectual disabilities live in the community [23]. Many of them also suffer from loneliness and feel deprived of the opportunity to live a decent life. Homecare assistants are employed to help with daily activities, but they rarely receive proper training for the job. They are expected to support the clients with regard to various matters, including household work, paying bills, picking up drugs from the pharmacy, and making appointments with doctors, social services professionals, and social insurance personnel. They are also expected to motivate passive clients, comfort sad clients, and calm anxious and psychotic clients. Consequently, they need to understand and handle clients with diverse and possibly severe and comorbid psychiatric disorders.

Frequently, users of mental health services are requested to change for the better and accept whatever assistance is offered. Instead, the situation requires a change in the professionals’ understanding and attitude about their clients and their poor living conditions.

### Objective

Education that increases knowledge and improves understanding is essential for professionals who encounter people with psychiatric impairments. Such education should be interesting, relevant, useful, and affordable and preferably result in heightened professional status. The overall aim of the Kognus 4-step Education Program was to create and launch a cost-effective, high-quality education program for large groups of professionals with different backgrounds. The project had a patient-centered approach. The project sought to enable professional advancement, with a focus on its usefulness for society and minimal administrative requirements. The aim of this report is to present the implementation and evaluation of the program.

## Methods

### Preparation

In Stockholm county, approximately 15,000 individuals are employed as homecare assistants and nurse assistants for people with psychiatric and cognitive disabilities. They mostly lack formal education within the field of psychiatry. The Swedish Board of Welfare specifically directed funding for projects that can improve education among this large group of healthcare personnel.

The gradual deterioration in the quality of care of people with psychiatric and intellectual disabilities prompted two Kognus promoters (i.e., SB, a senior cognitive behavioral therapy-trained psychiatrist/associate professor; AL, an administrator of education on psychiatric disabilities with a professional background in teaching and economics) to apply for funding. Because of the massive demand for useful and cost-effective education, which could not be achieved by the currently available academic curricula, a novel form of education was needed. Nine of the 14 city district administrations (each is independent from the others, and all are responsible for elderly care, support, and services for people with disabilities, urban work, and social psychiatry), five independent agencies (i.e., unit for the homeless, city job-seeking agency, Swedish psychiatry association for attendees, Swedish National Council for Alcohol and Drug Addiction, and Transcultural Health Center), and seven different client/patient associations (representing people with autism, attention-deficit/hyperactivity disorder [ADHD], obsessive-compulsive disorder, bipolar disorder, schizophrenia, addiction, and intellectual disability) supported the application for funding. Representatives from the different agencies formed a steering committee and a professional reference group. Representatives from the client organizations formed the non-professional reference group. In the discussions, municipal representatives, psychiatric care municipalities, and non-professional representatives participated. The content of all of the courses was agreed to be evidence-based and include recent research. Only highly ranked lecturers and clients with experience with living with psychiatric disability were invited to lecture. Moreover, every session was agreed to be carefully evaluated, and the content and lecturers could be modified based on the participants’ evaluations.

The process of forming the project included the following:

1. The Kognus promoters provided a paper for discussion of the educational content in consultation with spokesmen from the client associations.
2. The discussion paper was submitted to the professional reference group for their comments.
3. The revised paper was submitted to the steering committee for a decision.

Between 2009 and 2012, we developed and ran the Kognus Education Program. In 2013–2014, the Swedish Social Security Service and National Employment Agency administered the program.

### Participants

The organizers recruited participants through circulars that were distributed to various workplaces within the Stockholm municipality and county and by word of mouth. Personnel from a wide range of occupations, employed either by private companies or the municipality, participated. Homecare assistants represented the initial and largest group, but this group was gradually broadened to encompass many types of healthcare workers, social workers, and teachers. In 2013–2014, social security and employment agency employees also participated.

**Fig. 1:**
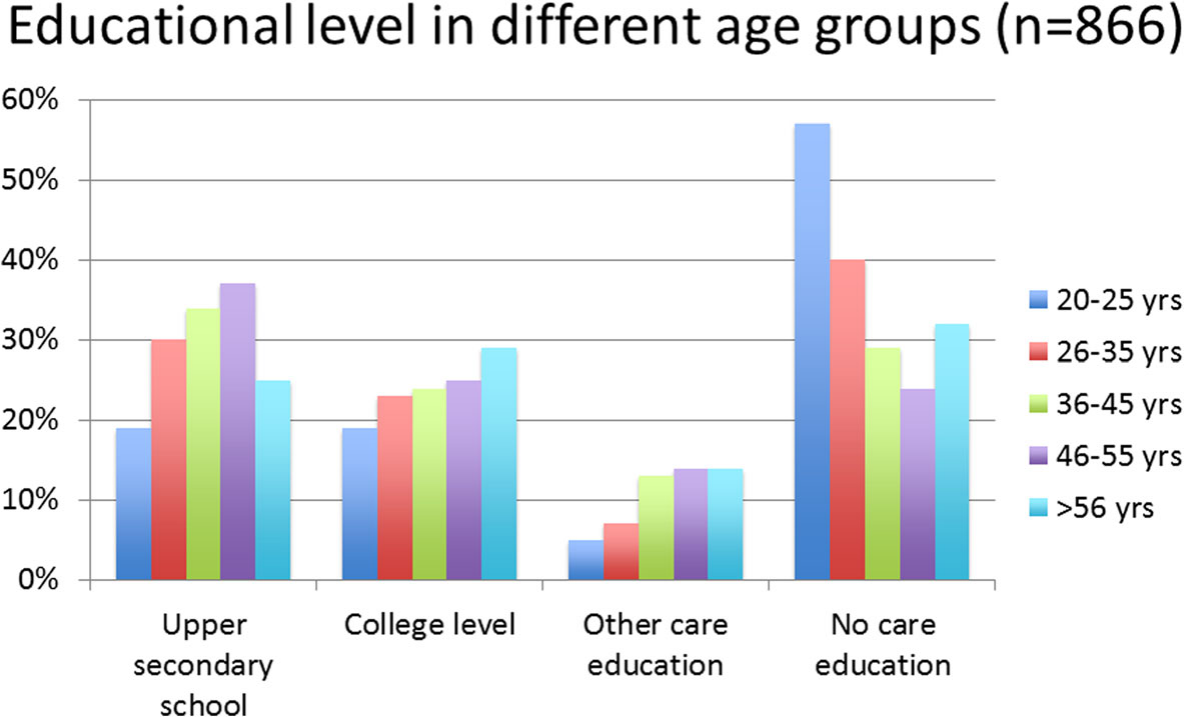
Education in different age groups

Evaluation data were obtained from a subgroup of 883 respondents who participated on-site in 2010 and 2012. Of these, 78% were female, and 82% spoke Swedish as their native language. Eighty percent were older than 35 years old, and 91% had completed at least 12 years of schooling (upper secondary school). The most common occupation involved caring for individuals with psychiatric and intellectual disabilities with regard to daily care and housing. Other job duties included social activities that were organized by community-based mental health services and working with job placement. A minority of the respondents (11%) worked within the psychiatric healthcare system, and 4% were school personnel. Among those who were younger than 36 years old, approximately 50%  lacked healthcare education. However, 25% of the participants had a college healthcare education, which implies a wide range of theoretical knowledge among the participants. The experience of caring is an asset in mental healthcare, but the lack of a formal education in the younger age groups is problematic. Figure 1 shows educational levels in the different age groups. Although the vast majority of the participants worked with clients with severe psychiatric problems, 49% never received counseling and were consequently left to solve very difficult situations themselves.

### Selection of lecturers

The lecturers were carefully selected based on expertise within their field (most of them were clinicians), experience in teaching, and ability to reach out. Approximately half of the lecturers had a PhD, and several were authors of textbooks. The lecturers were provided with a framework and title for their presentation and were informed that the focus should be on understanding the patients’ problems and behaviors rather than presenting statistics and academic details. The prerequisites for being hired were consent to be filmed and sharing educational material (e.g., PowerPoint handouts). The lecturers were paid $1365 for two or three 45-min lectures.

### Clients’ participation

Many of the sessions also included a talk by clients who were recruited from a client association or elsewhere to share his/her own experiences. Most of them were client association board members and lectured on a regular basis. Others wished to lecture and took the opportunity to do so. None of the lecturers was persuaded to lecture. For example, following the lecture on autism, a young woman with Asperger disorder read directly from a script in an inflexible and autistic way. She spoke about her difficult childhood, being an outsider, and being bullied endlessly throughout school. She also shared her great relief when she was finally admitted to a special class for students with Asperger disorder. In another session, a severely autistic and self-harming woman recited her own poignant poems to the audience. Another woman participated in the lecture on ADHD. She preferred a dialogue with the lecturer/psychiatrist rather than giving her own talk. This conversation began with selected citations directly from her medical record. She had been a challenge for her family and was an early dropout from school. Proper treatment had helped her to live a life that was based on her own priorities. In the session on intellectual disability, approximately eight individuals with intellectual disability took to the stage, accompanied by a single caregiver. They readily talked about their struggles in life and the ways in which they learned how to feel proud of themselves despite their cognitive impairments. One charming young woman with intellectual disability and William’s syndrome sang a beautiful song on stage. People with William’s syndrome tend to be musically talented. One person with obsessive-compulsive disorder explained how it felt to not be able to stop rituals because of extreme urges to get things “just right.” An elderly woman spoke about her outrageous behaviors during her manic episodes, which caused her own children to turn their back on her. A man in his 40s who had injected amphetamine for decades spoke about treatments and relapses and his hopes for new teeth and a drug-free future.

The participants were invited to ask questions. However, they were carefully instructed before the session to refrain from asking anything that they themselves would feel uncomfortable to respond to in front of an audience of hundreds of participants. Conversely, the clients were informed that there was no need to answer questions that they felt were uncomfortable or difficult in any way. The clients’ narratives were highly appreciated and often referred to as “mind changers” by the participants. The clients were paid approximately $100 for their 25–45 min participation.

### Web-based platform

We developed a web-based platform to allow enrollment with minimal administration. We also used the website to distribute information and provide useful links, reference lists, and handouts. A guidebook for caregivers and an instructive film (in both Swedish and English) on ways to help clients with autism spectrum disorder in their daily lives were produced within the project and made available on the website and on YouTube (https://www.youtube.com/watch?v=Lw6HZiHUixY). The website was also used to monitor the quality of each session. The participants anonymously graded the sessions online using a 5-point Likert scale, in which a score of 5 indicated “excellent.”

### Setting

Moderators introduced the speakers and led the discussion. The participants could ask questions directly in class or submit written questions on paper or via short message service (SMS; i.e., text message). Educational material, such as handouts, could be downloaded from the website after each session, but only after the participant had completed the evaluation on the quality of the session. All of the courses were provided free of charge.

### Control of attendance and examination procedures

Attendance was manually confirmed by each attendee’s signature in each session. To receive a certificate of attendance, the participants must have attended at least 75% of the sessions. In the final session, we administered a multiple-choice test in the lecture hall. To receive a diploma, a minimum of 70% correct responses on the multiple-choice test was required, in addition to attending the minimum number of sessions as described above.

### Description of educational interventions

**Fig. 2:**
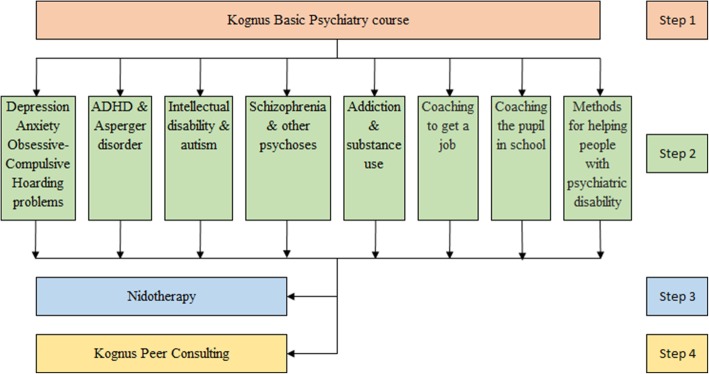
Kognus

The Kognus 4-Step Education Program consists of independent courses, including an 18-session basic weekly introductory course on psychiatric disability (on-site or online), eight advanced courses that were presented weekly for 8 weeks, and two highly specialized training programs (Nidotherapy and Peer Consultation, each with sessions that spanned over 1 year). The courses are shown in Fig. 2. We offered a biological explanation for most psychiatric symptoms and emphasized psychosocial and cultural complications. We labeled our approach “Biopsychosocial” and summarized our core values and ideas as the following:

1. Severe mental illness and disabilities primarily have a biological basis.
2. Mental illness and disability will always have social consequences.
3. Dignified care, support, and care require good empathy.
4. Approaches must be tailored to the recipient’s circumstances.

### Evaluation of the program

The participants evaluated the quality and meaningfulness of each session on the website. Other types of information were collected through questionnaires. These questions varied between the different courses because our objective was to gain a broad overview of the participants’ educational background, working conditions, views on the education program, and whether the education changed their ways of working. Distributing the questionnaires in class ensured that almost everyone completed them. Moreover, to obtain in-depth information on the ways in which the different courses were valued, groups of 5–6 volunteers (i.e., focus groups) from each educational course were invited to share their views. Revisions were then made based on the participants’ online evaluations, discussions in the reference groups, and comments from the focus groups. Proposals for major changes were submitted to the steering committee for a decision.

### Courses

#### Step 1. The Kognus basic psychiatry course

The first step in the Kognus 4-Step Education Program consisted of an 18-session Basic Psychiatry course that covered psychiatric problems, complications, and disorders. Each weekly session lasted 3.5 h (including breaks). The size of the groups varied between just above 100 to approximately 400 participants. The course did not seek to convey detailed knowledge about specific disorders but rather the consequences of the disability and a better understanding of the clients’ experiences and hopes. We also informed participants about novel research and important findings related to the topic. An expert (e.g., psychiatrist, psychologist, occupational therapist, social worker, special education teacher, or other professional) lectured. The questions from the participants to the lecturer were collected before a coffee break that allowed the lecturer to choose relevant questions and prepare answers. The topics that were covered in the sessions and lecturers’ professional backgrounds are presented in Table 1.

**Table 1:** The topics included in the 18 sessions Kognus Basic Psychiatry course and lecturers

Introduction to psychiatric disability	1. Aims and biopsychosocial outlook; What are psychiatric disability and psychiatric disorders? • ^1^Psychiatrist, PhD, psychotherapist
Autism spectrum disorder	2. What is autism spectrum disorder? • ^1^Psychiatrist, PhD, psychotherapist 3. Providing assistance to people with autism spectrum disorder in everyday life • ^2^CBT trained autism counsellor Poems on my life living with autism spectrum disorder • Person with Asperger disorder and self-harm 4. Stress in people with autism spectrum disorder • ^3^Special education teacher
Attention-Deficit/Hyperactivity disorders (ADHD)	5. What characterizes ADHD • ^1^Psychiatrist, PhD, psychotherapist My life with ADHD • Person with ADHD
Diet, health, depressive disorders and psychopharmacology	6. Healthy diet and psychiatric health Introduction to depressive disorders; Introduction to psychopharmacology • ^4^Psychiatrist, PhD
Intellectual disability and other subjects	7. Introduction to intellectual disability; Introduction to Nidotherapy and the Kognus Peer Consultation training course • ^1^Psychiatrist, PhD, psychotherapist; ^4^Psychiatrist, PhD; ^5^Teacher
Psychotic and bipolar disorders	8. Introduction to psychoses and bipolar disorder • ^4^Psychiatrist, PhD disorder Living with bipolar • Person with bipolar disorder
Psychosis and cognition	9. Cognitive impairment in people with psychotic disorders • ^5^Psychologist
Obsessive-compulsive disorder and chronic tic disorder	10. Obsessive compulsive disorder and tics • ^1^Psychiatrist, PhD, psychotherapist My life with obsessive-compulsive disorder and Tourette • Person with obsessive-compulsive disorder
Intellectual disability employment and aids for intellectual disability	11. Supported employment and cooperative jobs for people with intellectual disability • ^6^Social worker Living with intellectual disability • Individuals with intellectual disability Aids for cognitive disabilities • ^7^Occupational therapist
Brief therapy	12. How to work with brief solution-focused therapy • ^8^Author, autism counsellor
Cognitive behaviour therapy	13. Introduction to cognitive behaviour therapy • ^9^Psychologist, PhD
Alcohol/substance use related disorders	14. Substance abuse; My life as a drug addict • ^3^Psychiatrist, PhD • Person with drug addiction
Challenging behaviours	15. How to work with challenging behaviours • ^3^Special education teacher
Aids in interviews	16. Rating scales and structured interviews as aids • ^10^Psychiatrist, PhD
Legislation and transcultural psychiatry	17. Legislation and rights for people with psychiatric disability • ^5^Teacher Transcultural psychiatry • ^11^Psychiatrist, PhD
Networking and examination	18. Networking – how to succeed • ^5^Teacher What networking did for me • Person with psychiatric disability Written examination

Superscript numbers depict professional and academic status of the 11 lecturers and the clients with psychiatric disabilities

#### Online course

We used two models of online courses. The first model was tested in 2012. Five selected sessions from the Kognus Basic Psychiatry course were distributed online in real-time and in three different cities in Sweden (Malmö, Skellefteå, and Mölnlycke). Groups of 15 to several hundred participants attended online one or several of the lectures in lecture halls and cinemas. At each site, a moderator was responsible for the meeting, checking attendance, and communicating with the organizers in Stockholm. These online participants were also invited to send questions to the lecturer by SMS. Similar to the on-site participants, the online participants could print the handouts through the website.

The second model was used in 2013 and 2014 when *Consensio* ran the program (see below). The program delivered the full Kognus Basic Psychiatry course nationwide to employees of the social security and employment services. The sessions were conducted in real-time, but there were no special arrangements to convene groups of attendees. Instead, the participants followed the lectures on their individual computers or in smaller groups. By logging into the session, their attendance could be monitored. The sessions were available online for 2 weeks. Participants with at least 75% attendance at the sessions were invited to take the written examination.

#### Step 2. Advanced specialized courses

Participants who had attended the Kognus Basic Psychiatry course were invited to the second step in their education that consisted of a choice of eight different specialized courses (Fig. 2). Each course included 6–8 sessions, and each session lasted 3.5 h, including breaks. A written exam was optional. The topics that were covered in the sessions and lecturers’ backgrounds are presented in Additional file 1: Table S1a-h.

#### Step 3. Nidotherapy

The third step targeted limited groups for education on nidotherapy, a method that was developed by the British professor and community psychiatrist Peter Tyrer and seeks to change the environment to better suit the psychiatric patient [24, 25]. It includes an environmental analysis, finding ways to reach a better fit between the environment and the patient, and monitoring progress. Nidotherapy is not designed to change the client’s behavior but rather alter his/her physical and social environment with assistance from a nidotherapist. The task of the nidotherapist is to explore possible concrete areas that can be improved for the patient according to his/her wishes (i.e., not necessarily the wishes of the professionals). The nidotherapist assists in implementing these changes over approximately 9–12 months. Collaboration within the client’s network, to which the client consents, is crucial to achieve success. The client is an active partner to reach the selected and attainable goals.

The nidotherapy training course included nine 3-h sessions over a 12-month period. The participants represented a variety of professional backgrounds, such as working at psychiatric outpatient units, nongovernmental organizations, and employment services. No costs were involved. The first session of the course provided participants with a free textbook on nidotherapy [26]. In the second session, their knowledge on nidotherapy was examined in a multiple-choice test.

After the open introductory lecture on nidotherapy, a total of 25 professionals chose to participate in the nidotherapy training courses. Two nidotherapy trainees worked in conjunction with one client throughout the course. All of the clients had psychiatric disabilities and were recruited from the trainees’ workplaces. The potential clients could, if they desired, come to class and present himself/herself and the problem he/she wanted to solve. During the supervision sessions, the trainees jointly discussed ongoing work and thus acquired an understanding of ways to deal with different types of problems. Examples of problems included organizing the home, finding ways to finance and purchase a dishwasher, and accompanying a client who was afraid of vomiting on the bus. Notably, the nidotherapist trainee did not treat any psychiatric symptoms. At the end of the course, the trainees were examined with extensive written and oral examinations. Each participant received a score (ranging from 1 to 10) for each of the following five areas: (*i*) gaining trust, (*ii*) perseverance, (*iii*) flair (i.e., lateral thinking), (*iv*) pacing (i.e., moving forward at the right pace for the patient), and (*v*) collaboration.

The participants were awarded diplomas following successful examinations.

#### Step 4. Peer consultation training

The fourth step of the Kognus program is highly specialized education on peer consultation for a small, select group who had already attended the Kognus Basic Psychiatry course and two or more of the Advanced Specialized courses or equivalent education that was provided elsewhere.

We had learned that staff at the community-based mental health services, job centers, social security system services, and schools often felt helpless when asked to solve problematic situations around patients/clients/pupils with mental problems. Licensed counselors were rarely available because of the costs involved. Additionally, if the counselor is unfamiliar with the conditions and/or type of clients at the workplace, then the external counselor’s contribution to solving problems may be regarded as insufficient or pointless. The Peer Consultation Training course was developed to fill the gap when the staff’s own ability fails but when the magnitude of the problem is considered too small to justify a licensed counselor. We presumed that professionals, when trained in matters that are related to mental disability and the art of supervising others, would be able to guide colleagues with similar professional backgrounds as themselves. The model builds on the assumption that skilled workers develop a special cultural competence within their own field over the course of many years. This ability may provide a unique opportunity to grasp and find solutions.

The Peer Consultation Training course included 38 lectures that lasted 3 h over a 12-month period. The teachers were licensed counselors in cognitive behavioral therapy (CBT), autism spectrum disorder, and/or intellectual disability. The program educated and trained participants in how to find workable solutions to benefit the individual patient/client/pupil. These peer consultation trainees consulted with as many as three different clients and participated in and gave feedback on a fellow students’ supervision of another three clients. In all, 55 workplaces received 3–5 days of 90-min supervised sessions with a peer consultation trainee over a 9-month period. Topics for supervision included, for example, aggression, behavioral problems, addiction, mental illness, home or school/work issues, self-harm, worrying, hoarding, sleep disturbances, and food intake concerns.

The peer consultation trainees were carefully selected from a total of 146 applicants who were recruited from the Kognus courses. To be accepted to the peer consultation training program, the participants needed to have at least 5 years of experience within their own profession. Moreover, before acceptance, they were invited to a group interview to evaluate their suitability to become peer counselors. They were jointly introduced to a couple of written case reports on a person with psychiatric problems who faced a difficult social situation. They were asked to discuss ways to deal with the situation. Based on each applicant’s ability to understand the situation, listen, and propose convincing and wise solutions gently to others, the assessors independently scored their suitability for becoming a peer counselor and agreed on which of them to accept.

The peer consultation lecturers were licensed counselors in CBT and/or autism spectrum disorder with intellectual disability (*n* = 4), a teacher who was trained in autism (*n* = 1), a psychiatrist (*n* = 1), a lawyer who was trained in patients’ rights (*n* = 1), and a client with self-harm behavior and autism (*n* = 1). The required homework included reading books on psychiatric disabilities and written assignments. The topics that were covered in the sessions are presented in Additional file 2: Table S2.

## Results

### Evaluation of step 1: basic psychiatry course

Of the on-site participants, 72% had satisfactorily attended, and 43% received a diploma, which required participating in at least 75% of the sessions and successfully passing an optional written exam. Online education is known to have a high dropout rate [27]. In our setting, only 11% of the nearly 5000 online participants attended the online course satisfactorily. The on-site Kognus Basic Psychiatry course in 2010 and 2012 received a mean rating of 4.4 on a 5.0 scale. After completing the course, 90% agreed that the course was good or very good. Nearly all of the respondents were willing to recommend the course to others, and only 0.2% of the participants were unwilling to recommend it. A total of 97% reported a substantially greater understanding of people with psychiatric disorders upon completion of the course, and 91% of the participants reported that a better understanding led to changes in their methods of working. A total of 46% of the respondents agreed that they felt much more confident in their jobs following the education course.

### Evaluation of step 2: the advanced specialized courses

**Fig. 3:**
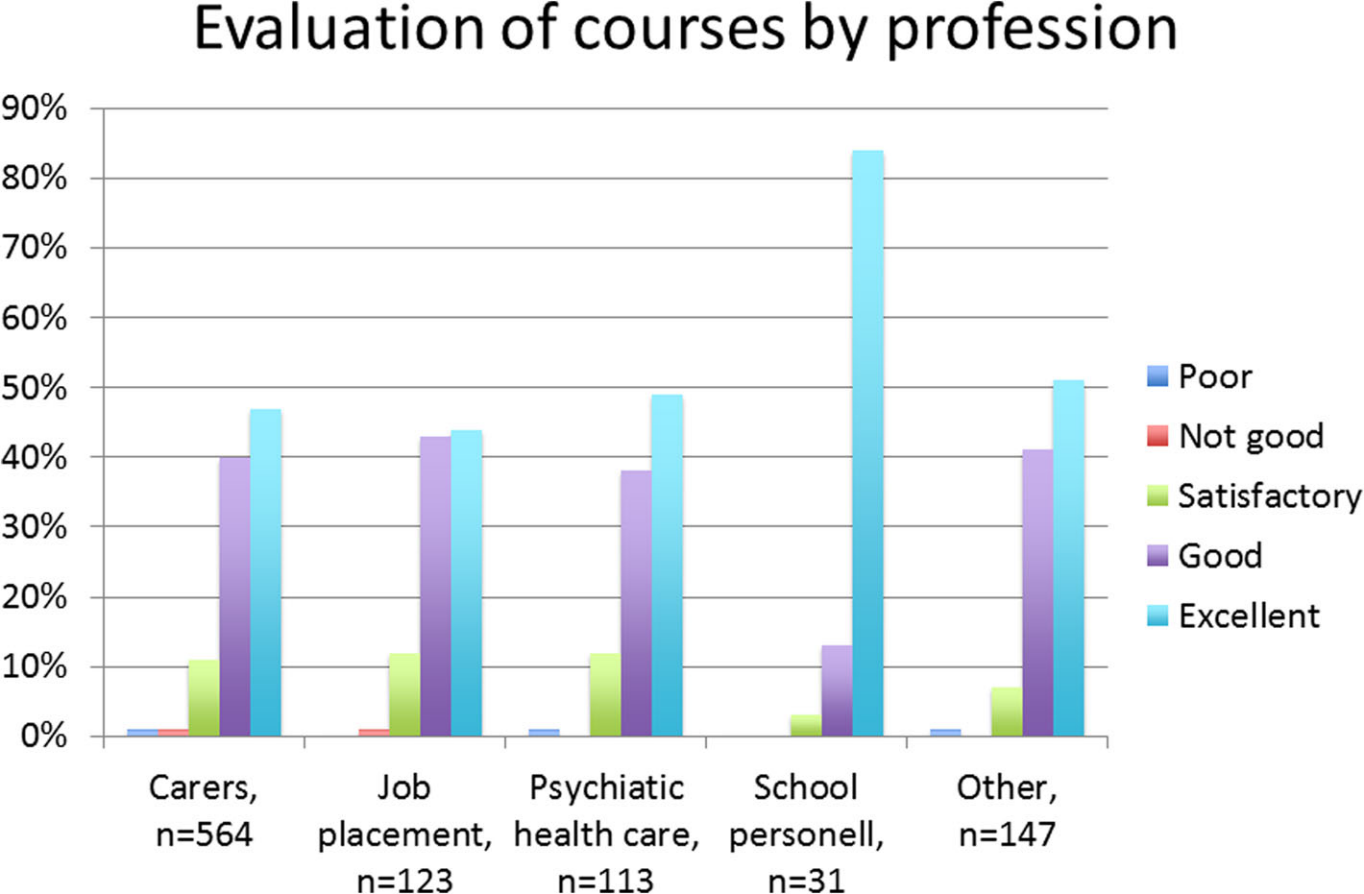
evaluation by profession

In the advanced courses, 82% had > 75% attendance, and 34% passed the written exam. The Advanced Specialized courses received a mean rating of 4.3 on a 5-point Likert scale by a subset of 605 participants who attended courses in 2010–2012. A total of 80% reported that they would highly recommend the courses to others, and 18% agreed with this statement to some extent. Evaluations of the step 1 and 2 courses, divided by profession, are presented in Fig. 3.

### Evaluation of step 3: Nidotherapy

All of the nidotherapy trainees reported that they wanted to continue to deliver nidotherapy in the future. Thirty-three participants in the Nidotherapy course (92%) passed the exams and received diplomas. However, we also wanted to perform a quality check of the ways in which the clients experienced nidotherapy. The clients were asked (by their nidotherapist trainees) if they were willing to anonymously answer a few questions over the phone on their experience of receiving nidotherapy. A total of 10 of 13 clients gave their permission. Nine clients were satisfied with receiving nidotherapy and would gladly recommend it to others, whereas one client was doubtful.

### Evaluation of step 4: peer consultation training

Of the 52 interviewed applicants, a total of 21 were selected for the classes, and 19 peer consultation trainees received diplomas. Their professions included special education teachers (*n* = 5), administrators at employment services (*n* = 2), and home-care assistants or assistants for the intellectually disabled (*n* = 12). The peer counselors rated the quality of their education as 4.7 on a 5-step Likert scale.

The supervision was evaluated through telephone interviews with the managers at all of the workplaces. Both the model that consisted of peer consultation and the work of each consultation trainee were highly appreciated. The model received a score of 4.7 on a 5-point Likert scale, and the trainees received a mean score of 4.8. Overall, 49 of the 55 participating workplaces (89%) reported that they wished to continue the peer consultation.

For an overview of the number of courses, number of attendees, attendance rates, and written examinations, see Table 2.

**Table 2:** Participants in the Kognus 4-step Education Program, participants with ≥75% attendance and participants receiving diploma (> 75% attendance and passing written exam)

Step 1: Basic course	Basic psychiatry on site and online courses, provider	Number of admissions	≥75% attendance	Diploma n (%)
	On site 2009–2012, Kognus own regime	1415	73%	556 (39%)
	On site 2013–2014, other provider	696	70%	348 (50%)
	Online 2013–2014, other provider	4480	11%	58 (1%)
Step 2: Advanced courses	On site 2009–2012, Kognus, own regime	1375	80%	456 (33%)
	On site 2013–2014, other provider	947	84%	341 (36%)
Step 3 and Step 4: Nidotherapy and Peer Consultation	On site 2012–2013 Kognus, own regime	46	97%	42 (93%)

### Cost per participant

The fixed costs for the project were $276,000 per year, including three full-time employees and facilities for staff. The direct costs for the 18-session Kognus Basic Psychiatry course were $213 per participant, excluding facility rental. The direct costs for the Advanced Specialized courses were $106, excluding rentals. Each online presentation cost $3519. The Nidotherapy course cost $2013 per trainee (based on groups of 10 participants), and the Peer Consultation course cost $3838 per trainee (based on 10 participants). Comparable courses on psychiatry, available for psychiatric staff in Sweden, are much briefer and more costly. Attending a 2-day course (approximately 13 h) on psychiatry costs $500 to $1125, excluding facility rental. That is less than one-third of the lecturing hours that are provided by the Kognus basic course, and to a considerably higher cost.

### Generalizability of the Kognus 4-step education program to other professional groups

In 2013–2014, The Swedish Social Security Service and National Employment Agency received funding from the European Social Fund to start *Consensio*, a strategic education project that focuses on psychiatric disability and is aimed at their personnel. By implementing the Kognus 4-Step Education Program over an 18-month period, large groups of professionals without any basic education in psychiatry could receive such education. A total of three Kognus Basic Psychiatry courses, 11 advanced specialized courses, one Nidotherapy course, and one Peer Consultation Training course were launched on site, and two Basic Psychiatry courses were distributed online. *Consensio* only evaluated one of the Basic Psychiatry courses. This was done online and had a response rate of 58%. The course received a mean rating of 4.6 on a 5-point Likert scale. The results imply that the Kognus program was highly appreciated by other professional groups beyond those for whom it was originally designed.

The participants encouraged others to apply for the courses; thus, advertising the program was unnecessary. When the education program was closed in 2014 because of a loss of funding, we had considerable waiting lists for all of our Kognus courses.

## Discussion

The Kognus 4-Step Education Program was developed to meet the needs of various professional groups who seek to help people with mental disabilities. Over a 5-year period, a total of 4443 participants attended the courses on-site in Stockholm. Approximately 4480 professionals attended the Kognus Basic Psychiatry course online. We delivered advanced psychiatric courses that covered several psychiatric disorders, in addition to courses that taught methods to enable coaching to find jobs and manage school situations for pupils with psychiatric disabilities. Moreover, we offered Nidotherapy and Peer Consultation Training courses. Nidotherapy training had not been applied outside Great Britain before our program, and the Peer Consultation Training course was a new educational approach that was developed by us. In the Nidotherapy and Peer Consultation Training courses, nearly all of the participants passed thorough examinations.

The participants who attended the Kognus Basic and Advanced Education courses represented a wide range of professions, with different levels of education in mental health. The courses received top evaluations, and attendance was high. Almost all of the participants reported that they would gladly recommend the courses to others.

Virtually all of the participants stated that their own understanding of their clients had increased considerably after completing the Kognus Basic Psychiatry course. They also felt more confident at work, indicating an improvement in working conditions. Working conditions and routines are often difficult to change. There is limited evidence of the impact of patient involvement on healthcare practices [28], but 30% of the respondents experienced positive changes in this area. Education with patient involvement has been shown to promote self-reflection on attitudes and assumptions [29]. Other benefits were shown to include higher self-rated measures of comfort, confidence, and competence compared with controls [30]. The most important overall lesson learned was the powerful impact of the real-life dimension on interactions with patients.

Representatives of client organizations were involved in all aspects of planning and delivering the Kognus program, suggesting a solid partnership whereby patients can make meaningful, valued contributions [31]. The importance of an evolving role of patients as interdependent partners and co-producers in modern healthcare and the need for medical education research in this particular context have been stressed in recent research [32].

### Attendance and exams

In contrast to the high rates of attendance for the on-site courses, few participants who attended the online courses participated to a satisfactory extent. This can be explained by the fact that these participants were working across the country and often watched individually or in smaller groups and thus may have felt less obligated to attend the full course. Additionally, the motivation to attend the online courses may have come from the participants’ employers rather than the participants themselves. The high dropout rate could also be explained by known factors that predict dropout, such as low satisfaction with Internet courses and a lack of time to invest in completing the course [32]. Dropout rates were also shown to be higher among participants with a lower or middle educational level [33] (relevant for most participants in Kognus) compared with highly educated participants. On-site participants in Stockholm were asked during the final session if they would have preferred on-site or online courses. Of these participants, 74% preferred the on-site course in the lecture hall, 22% had no opinion, and only 4% preferred an online course. We believe that participating in a class with others, away from the regular work place, is crucial for reaching high rates of attendance, especially for people with little training in self-education. When participants gather in lecture halls (i.e., away from their work places) with a local on-site moderator, higher attendance rates can be expected. This is consistent with our own experience with our first attempt to deliver the course online in 2012. The attendance rates were communicated to be good when we provided a selection of online lectures to groups of professionals that were accompanied by a moderator on site.

Taking the exam was not mandatory, but it was necessary to receive a diploma. Between 39 and 50% of the participants in the basic course and approximately one-third of the participants in the advanced courses received a diploma. Over the years, there was a trend toward more participants taking the exams, albeit still fewer in the advanced courses than in the basic course. A possible explanation could be group pressure in the large basic course classes, and a basic course diploma could be perceived as more valuable than the advanced course diploma because of the basic course’s comprehensive content and long duration. The lack of financial incentives and new career opportunities are additional plausible reasons for not taking the examination. However, 10 of 19 participants who received diplomas from the final fourth step in the Kognus program (Peer Consultation Training course) had changed jobs and advanced in their careers 1 year after completion.

### A wider perspective

The Kognus 4-Step Basic Education Program was originally developed for homecare assistants with relatively low levels of education, but it proved to as useful and appreciated by more highly educated participants (e.g., working in job placement and social security services generally requires a college degree).

Factors that likely contributed to the large numbers of people who chose to attend our courses were the following: engaging and clinically relevant lectures that were free of charge, clients who were willing to convey their own experiences, and the integration of recent and thought-provoking research in the lectures. Altogether, these attributes of the program appeared to provide the participants a sense of what it means to suffer from mental disability. The program also allowed the participants to reflect on how they can change their own ways of working. From the perspective of working environments, the evaluations indicated that the program gave the participants insights that made their work more meaningful and gave them stronger professional self-confidence. The team behind the Kognus project comprised only a handful of people, which enabled flexibility and kept costs down. A possible future development would be to further tailor the courses for individual needs of various types of professionals, similar to what we did for teachers and job placement personnel in the Kognus advanced specialized courses (see Fig. 2). However, our initial overall ambition was to provide a common ground for professionals to learn about the nature and complications of psychiatric illness before delving into further specialization in specific areas. Another possible development of the program would be to invite patients and families to participate in the education.

### How was the initiative received, and why was it not supported more fully?

According to the head of the department for promoting psychiatric welfare in Sweden (The Swedish Association of Local Authorities and Regions [SKL]), the Kognus program was not a priority for further funding. The SKL is an employers’ organization that is charged with representing local governments. All of Sweden’s municipalities, county councils, and regions are members of SKL. The SKL’s representative (a lawyer by profession) concluded that local education providers throughout Sweden can themselves develop educational programs on mental health if needed. However, such development and implementation have not happened yet. Sweden has approximately 200,000 healthcare workers with little knowledge about how to approach and understand individuals with severe mental health problems.

The Kognus program was developed for participants with different levels of schooling and education and various professional backgrounds. Because of its flexibility, the program did not fit into established educational curricula. The Kognus program continuously adapts to the needs and requirements of participants, stakeholders, and contemporary research findings rather than adheres to legislative frameworks and bureaucracy. These were both strengths and weaknesses with regard to survival of the program. Another important shortcoming was our inability to market the program toward local politicians when SKL had denied further funding. Marketing is likely crucial for receiving financial support when a project, such as the Kognus program, does not nearly fit into a regular educational curriculum or traditional research topic.

### Limitations

Unfortunately, data collection was limited to only a subset of courses. Furthermore, we did not gather information about whether the program increased participants’ satisfaction with their jobs or enabled career advancement. We also do not know the reasons why participants did not complete the courses. Importantly, we do not know whether the participants’ clients benefited from the education, whether it affected their quality of life, or whether the program reduced mortality rates. It would have been a major undertaking to contact all of the clients of all of the participants, for which ethics approval would have been required.

We are unaware of any adverse events that occurred as a result of participating in the Kognus program because we did not ask the participants or lecturers about such adverse events.

### Conclusion

In summary, personnel without appropriate education who work with people with psychiatric or intellectual disabilities can be educated in large numbers at relatively low cost. Future research should focus on clients’ perspectives. It is crucial to learn whether an education program such as ours can improve clients’ quality of life and not only satisfy the needs of personnel.

## Additional files

Additional file 1: **Tables S1a-h.** Step 2: Kognus Advanced Specialized courses included in the Kognus 4-Step Education Program. Superscript numbers depict the profession/position of the lecturers. (DOCX 32 kb)

Additional file 2: **Table S2.** Content of the Peer Consultation course. (DOCX 21 kb)

## Abbreviations

ADHD: Attention deficit hyperactivity disorder
CBT: Cognitive behavioral therapy
