# Supplementary material for: Teaching psychiatry to large groups in society
Source: BMC Med Educ. 2019 May 16;19:148. doi: 10.1186/s12909-019-1596-9 (PMC6524333; doi:10.1186/s12909-019-1596-9)
Supplement: Supplementary file 1 — Tables S1a-h. Step 2: Kognus Advanced Specialized courses included in the Kognus 4-Step Education Program. Superscript numbers depict the profession/position of the lecturers. (DOCX 32 kb) [file 12909_2019_1596_MOESM1_ESM.docx]

**Additional file 1: Tables S1a-h.**

Step 2: Kognus Advanced Specialized courses included in the Kognus 4-Step Education Program. Superscript numbers depict the profession/position of the lecturers.

| **1a. Depression, Anxiety, Obsessive-Compulsive and Hoarding problems** | | |
| --- | --- | --- |
| **Session** | **Lecturers’ profession** | **Content of session** |
| 1 | - ^1^Psychiatrist, PhD, psychotherapist | - Facts on anxiety and depression, social anxiety & panic disorder |
| 2 | - ^1^Psychiatrist, PhD, psychotherapist - ^2^Spokes person from the Swedish OCD foundation | - Facts on obsessive-compulsive disorder (OCD) |
| 3 | - ^3^Psychiatrist | - Affective disorder and measures for support |
| 4 | - ^4^CBT psychologist, PhD | - CBT methods for OCD and panic disorder |
| 5 | - ^5^CBT psychologist | - Emotional instable personality, part I |
| 6 | - ^6^Psychiatrist, PhD, psychotherapist | - Emotional instable personality, part II |
| 7 | - ^7^CBT psychologist | - Mindfulness |
| 8 | - ^8^CBT psychologist, PhD | - Eating disorders - too much or too little |

| **1b. ADHD and Asperger disorder** | | |
| --- | --- | --- |
| **Session** | **Lecturers’ profession** | **Content of session** |
| 1 | - ^1^Psychiatrist, PhD, psychotherapist | - On what grounds do we set psychiatric diagnoses |
| 2 | - ^1^Psychiatrist, PhD, psychotherapist | - ADHD from within |
| 3 | - ^1^Psychiatrist, PhD, psychotherapist | - Asperger disorder from within |
| 4 | - ^2^Psychologist | - CBT for ADHD and self-help programs |
| 5 | - ^3^Social worker - ^1^Psychiatrist - ^4^Psychiatry nurse | - Education programs for ADHD - Stress management |
| 6 | - ^5^Coach for people with ADHD and autism | - ADHD and Asperger - What can a housing supporter do? |
| 7 | - ^6^Occupational therapist | - What aids can be helpful? |
| 8 | - ^7^Social worker - ^8^Teacher | - Ethics and legislation - Collaboration and networking |

| **1c. Intellectually disability and Autism** | | |
| --- | --- | --- |
| **Session** | **Lecturers’ profession** | **Content of session** |
| 1 | - ^1^Special education teacher | - Autism- an introduction |
| 2 | - ^1^Parent | - A parent’s experiences |
| 3 | - ^3^Special education teacher | - Intellectual disability |
| 4 | - ^1^Special education teacher | - The TEACCH Autism Program |
| 5 | - ^4^Special education teacher | - Communication with people with intellectual disability |
| 6 | - ^5^Psychiatrist | - Psychiatric disorders and intellectual disability |
| 7 | - ^1^Special education teacher | - Problematic behaviors. CBT and Behavior analysis methods for autism |
| 8 | - ^6^Teacher | - Ethics, legislation and collaboration |

| **1d. Schizophrenia and other psychoses** | | |
| --- | --- | --- |
| **Session** | **Lecturers’ profession** | **Content of session** |
| 1 | - ^1^Psychiatrist - ^2^Person with schizophrenia | - What is schizophrenia and other psychoses. - The patient perspective |
| 2 | - ^3^Psychologist | - An independent life |
| 3 | - ^4^Psychiatrist, PhD | - Integrated psychiatry and the importance of work alliance |
| 4 | - ^5^Psychologist | - Case management, part I |
| 5 | - ^6^Psychiatrist, PhD ^7^Spokesperson from the Swedish schizophrenia society | - Methods for employment - Life long learning; continuing school instead of employment |
| 6 | - ^5^Psychologist | - Case management, part II |
| 7 | - ^8^Psychologist, writer | - Cognition and schizophrenia |
| 8 | - ^9^Psychologist, text book author | - How to lead an independent life |

| **1e. Addiction and Substance use** | | |
| --- | --- | --- |
| **Session** | **Lecturers’ profession** | **Content of session** |
| 1 | - ^1^Textbook author | - Addiction- the kidnapped brain |
| 2 | - ^2^Psychiatrist, PhD | - Abuse and addiction among young people |
| 3 | - ^3^Psychologist, PhD | - Cannabis |
| 4 | - ^4^Social worker - ^5^Social worker, PhD | - Collaboration and network of addiction - Homelessness and addiction |
| 5 | - ^5^Psychologist, PhD | - Treatment for addiction |
| 6 | - ^7^Psychiatrist, psychotherapist - ^8^Psychotherapist | - CBT-based approach to co-addiction - Offspring of addicts |
| 7 | - ^9^Psychologist | - Introduction to Motivational interviewing (MI) |
| 8 | - ^10^Psychologist | - To handle conflict situations with the client |

| **1f. Coaching to get a job - for people with psychiatric disabilities** | | |
| --- | --- | --- |
| **Session** | **Lecturers’ profession** | **Content of session** |
| 1 | - ^1^Psychologist | - Techniques for fruitful conversations |
| 2 | - ^2^Psychiatrist, PhD, psychotherapist | - BOS -A questionnaire to support finding suitable work for people with psychiatric disability |
| 3 | - ^3^Autism consultant - ^4^Person with Asperger disorder | - Communication between the coach and the client |
| 4 | - ^5^Work Consultant - ^6^Teacher | - How to sit on your hands - To chart skills and jointly find solutions to a problem |
| 5 | - ^7^Project manager with physical disability | - To "sell" the client into a workplace |
| 6 | - ^8^Workplace supervisor | - Coaching the client at the workplace |
| 7 | - ^6^Teacher - ^9^Person with a psychiatric disability | - Ethics, legislation - Collaboration |

| **1g. Coaching the pupil in school - youth with psychiatric disabilities** | | |
| --- | --- | --- |
| **Session** | **Lecturers’ profession** | **Content of session** |
| 1 | - ^1^Psychiatrist, PhD, psychotherapist | - Psychiatric impairment in children and teenagers |
| 2 | - ^1^Psychiatrist, PhD, psychotherapist - ^2^Parent with ADHD | - Bullying and violation - To inform parents about disabilities |
| 3 | - ^3^Special education teacher | - Gross motor skills difficulties and dyslexia |
| 4 | - ^4^Special education teacher, PhD | - An inclusive school |
| 5 | - ^5^Special education teacher | - Stress and problem-creating behavior, part I |
| 6 | - ^5^Special education teacher | - Stress and problem-creating behavior, part II |
| 7 | - ^3^Special education teacher | - Measures for pupils with learning disabilities |
| 8 | - ^6^Teacher | - Skills needed in today's school |

| **1h. Methods for helping people with psychiatric disability** | | |
| --- | --- | --- |
| **Session** | **Lecturers’ profession** | **Content of session** |
| 1 | - ^1^Author, autism counselor | - The professional role and methods |
| 2 | - ^2^Special education teacher - ^3^Special education teacher | - A solution-focused approach, part I |
| 3 | - ^2^Special education teacher - ^3^Special education teacher | - A solution-focused approach, part II |
| 4 | - ^4^Psychologist | - Introduction to cognitive behavior therapy |
| 5 | - ^5^Special education teacher - ^6^Parent to children with autism | - Social stories |
| 6 | - ^1^Psychiatrist, PhD, psychotherapist - ^8^Teacher | - Nidotherapy - The Cat-kit: facilitated communication in autism |
