# Supplementary material for: Teaching psychiatry to large groups in society
Source: BMC Med Educ. 2019 May 16;19:148. doi: 10.1186/s12909-019-1596-9 (PMC6524333; doi:10.1186/s12909-019-1596-9)
Supplement: Supplementary file 2 — Table S2. Content of the Peer Consultation course. (DOCX 21 kb) [file 12909_2019_1596_MOESM2_ESM.docx]

**Additional file 2: Table S2.** Content of the Peer Consultation course

| **Session** | **Date** | **Hours** | **Lecturers’ profession** | **Content of session** |
| --- | --- | --- | --- | --- |
| 1 | 29-Aug | 08.30-12.00 | Teacher | Introduction |
| 2 | 30-Aug | 08.30-12.00 | CBT psychologist and Autism consultant | Different perspectives on the client’s situation: case discussion |
| 3 | 05-Sep | 08.30-16.30 | CBT psychologist | The workplace staff/group processes |
| 4 | 06-Sep | 08.30-16.30 | CBT psychologist | The workplace staff/group processes |
| 5 | 12-Sep | 08.30-16.30 | Teacher | Group exercises: Different methods for asking for description of a problem |
| 6 | 13-Sep | 08.30-16.30 | CBT psychologist | The workplace staff/group processes |
| 7 | 19-Sep | 08.30-12.00 | CBT psychologist | The workplace staff/group processes |
| 8 | 20-Sep | 08.30-12.00 | CBT psychologist | Communication |
| 9 | 26-Sep | 08.30-16.30 | CBT psychologist | Active listening |
| 10 | 27-Sep | 08.30-16.30 | CBT psychologist | Anxiety-related problems |
| 11 | 03-Oct | 08.30-12.00 | CBT psychologist | Anxiety-related problems |
| 12 | 04-Oct | 08.30-12.00 | CBT psychologist | Anxiety-related problems |
| 13 | 10-Oct | 08.30-12.00 | Teacher | Group exercises: Motivational interviewing |
| 14 | 11-Oct | 08.30-12.00 | CBT psychologist | Supervising the counseling |
| 15 | 17-Oct | 08.30-12.00 | Autism consultant | Autism and intellectual disability |
| 16 | 18-Oct | 08.30-12.00 | Autism consultant | Autism and intellectual disability |
| 17 | 24-Oct | 08.30-12.00 | Autism consultant | Autism and intellectual disability |
| 18 | 25-Oct | 08.30-12.00 | Autism consultant | Supervising the counseling |
| 19 | 07-Nov | 08.30-12.00 | CBT psychologist | Obsessive and compulsive behaviors |
| 20 | 08-Nov | 08.30-12.00 | CBT psychologist | Obsessive and compulsive behaviors |
| 21 | 14-Nov | 08.30-12.00 | CBT psychologist | Obsessive and compulsive behaviors |
| 22 | 15-Nov | 08.30-12.00 | CBT psychologist | Obsessive and compulsive behaviors |
| 23 | 21-Nov | 08.30-12.00 | Autism consultant and  CBT psychologist | Supervising the counseling |
| 24 | 22-Nov | 08.30-12.00 | Autism consultant | Autism and intellectual disability |
| 25 | 28-Nov | 08.30-12.00 | CBT psychologist | Tutorials on tutorials |
| 26 | 29-Nov | 08.30-12.00 | Lawyer | Autism and intellectual disability |
| 27 | 05-Dec | 08.30-12.00 | Autism consultant | Tutorials on tutorials |
| 28 | 06-Dec | 08.30-12.00 | Teacher | Legislation, ethical issues |
| 29 | 12-Dec | 08.30-12.00 | Lawyer | Cases |
| 30 | 13-Dec | 08.30-12.00 | Autism consultant and CBT psychologist | Supervising the counseling |
| 31 | 19-Dec | 08.30-12.00 | CBT psychologist | Supervising the counseling |
| 32 | 20-Dec | 08.30-12.00 | Autism consultant | Autism and intellectual disability |
| 33 | 17-Jan | 08.30-12.00 | Autism consultant | Tutorials on tutorials |
| 34 | 24-Jan | 08.30-12.00 | Autism consultant and  CBT psychologist | Supervising the counseling |
| 35 | 14-Feb | 08.30-12.00 | Psychiatrist | Evaluation |
| 36 | 28-Feb | 08.30-12.00 | CBT psychologist | Supervising the counseling |
| 37 | 28-Mar | 08.30-12.00 | Autism consultant | Supervising the counseling |
| 38 | 15-Apri | 08.30-12.00 | Autism consultant | Follow-up and comparisons of the prior perspectives on the case discussion from session 2.  Conclusions |
